# Supplementary figures and images for: Individuals co-exposed to sand fly saliva and filarial parasites exhibit altered monocyte function
Source: PLoS Negl Trop Dis. 2021 Jun 9;15(6):e0009448. doi: 10.1371/journal.pntd.0009448 (PMC8189443; doi:10.1371/journal.pntd.0009448)

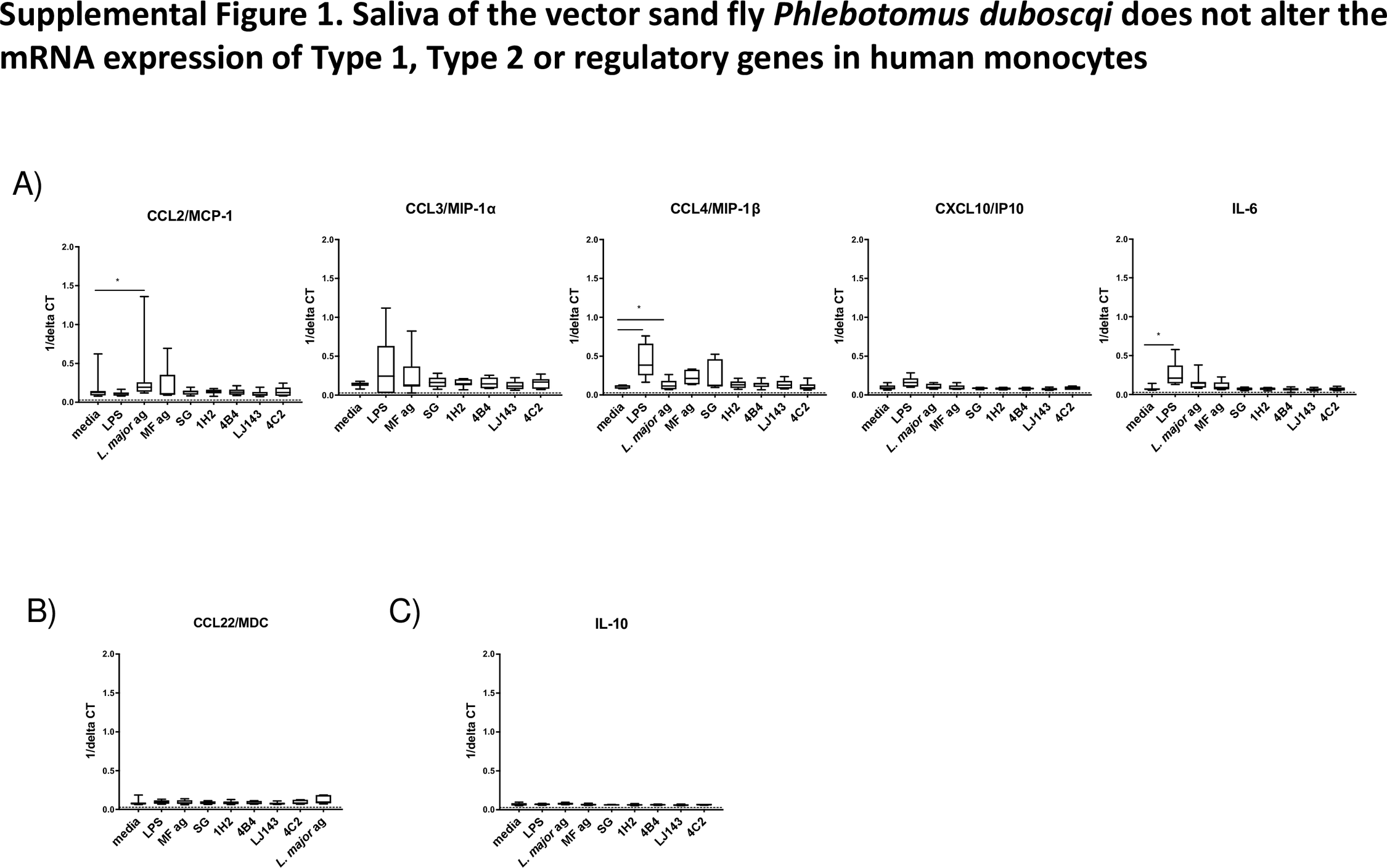

Supplement: S1 Fig — Human monocytes were unexposed (media) or exposed to either SG Pd, Leish ag, MF ag, LPS, or recombinant proteins 4B4, 1H2, LJ143, or 4C2 for 4 hours. mRNA expression of (A) Type-1 associated genes CCL2, CCL3, CCL4, IP10, and IL-6, (B) Type-2 associated gene CCL22, and (C) regulatory gene IL-10 was measured. Data are represented as box and whiskers (min to max) of 1/average ΔCt (n = 5–7). The mRNA detection threshold (dotted line) is 0.027. Statistical differences in mRNA expression are measured using Wilcoxon matched pairs signed-rank test; * (p<0.05). (TIFF) [file pntd.0009448.s001.tiff]

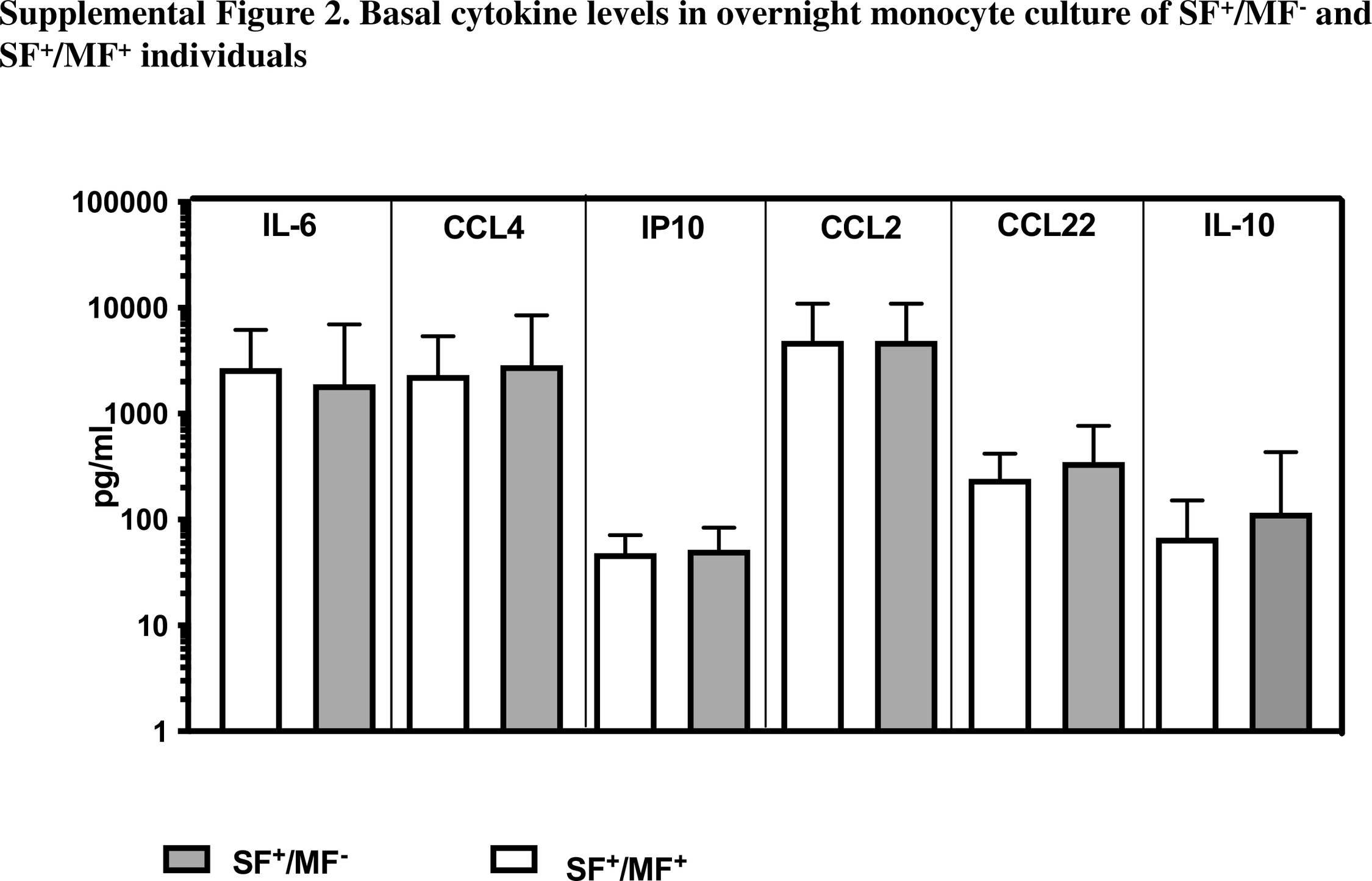

Supplement: S2 Fig — Monocytes were isolated from PBMC of SF+ /MF- (n = 13) and SF+ /MF+ (n = 11) individuals. Cells were cultured in media for 24 hours and supernatant was collected and cytokine levels were measured by Luminex. Bars represent the geometric mean with 95% CI. Statistical analysis was done using nonparametric Mann- Whitney test. (TIFF) [file pntd.0009448.s002.tiff]

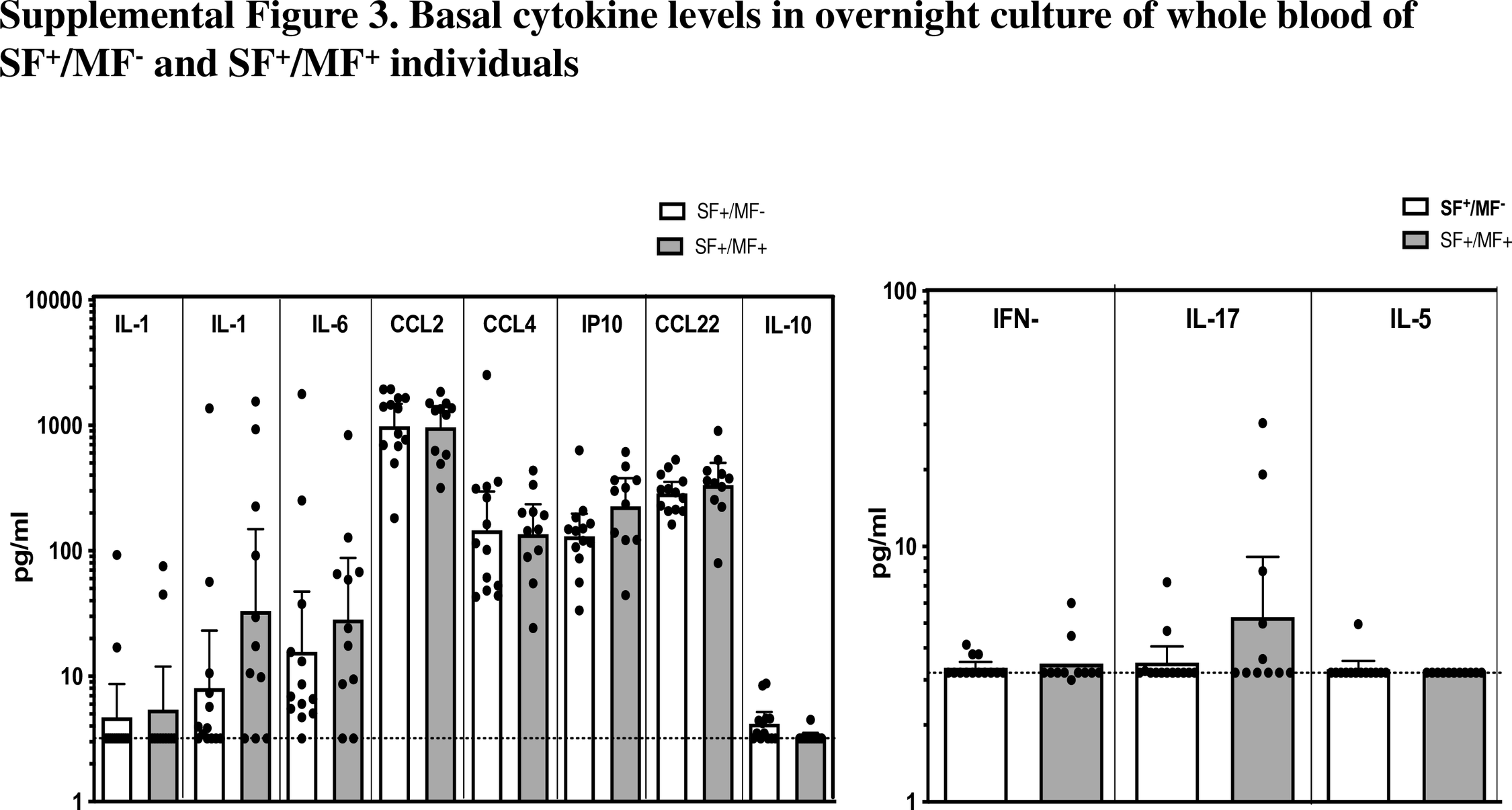

Supplement: S3 Fig — Whole blood from SF+ /MF- (n = 13) and SF+ /MF+ (n = 11) individuals were RBC-lysed and cultured in media for 24 hours. The supernatant was collected and cytokine levels were measured by Luminex. Bars represent the geometric mean with 95% CI. (TIFF) [file pntd.0009448.s003.tiff]

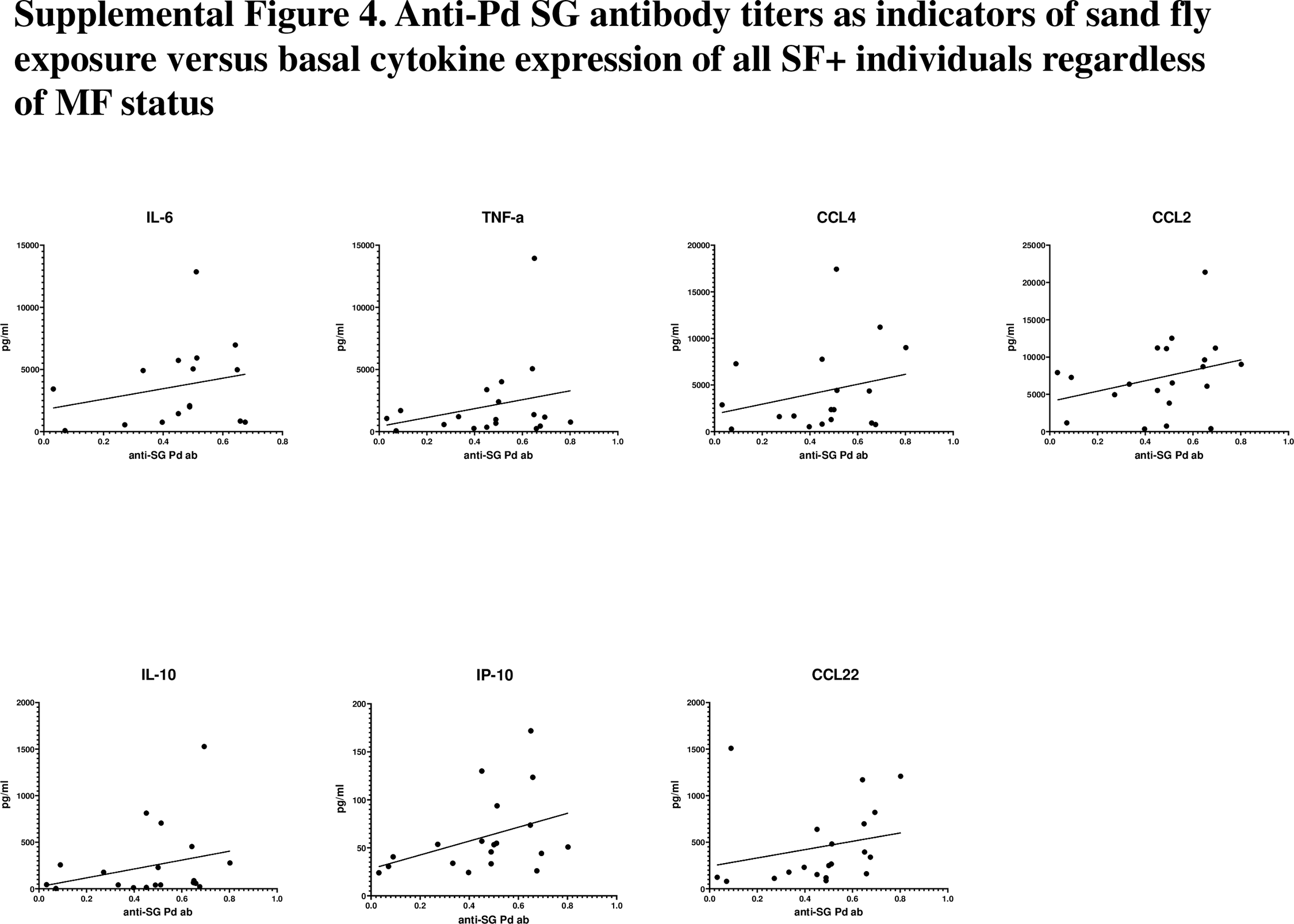

Supplement: S4 Fig — Our data suggests that there is no significant correlation between any of the tested cytokines and antibody titers. Nevertheless, the cellular immune response may be very different from the antibody response. (TIFF) [file pntd.0009448.s004.tiff]
