# Supplementary material for: Individuals co-exposed to sand fly saliva and filarial parasites exhibit altered monocyte function
Source: PLoS Negl Trop Dis. 2021 Jun 9;15(6):e0009448. doi: 10.1371/journal.pntd.0009448 (PMC8189443; doi:10.1371/journal.pntd.0009448)
Supplement: Attachment — Submitted filename: Manuscript_final_Review.docx [file pntd.0009448.s005.docx]

**Titles**

**Full title:** Individuals co-exposed to sand fly saliva and filarial parasites exhibit altered monocyte function

**Running title:** Sand fly bites and filaria alter APCs

**Authors’s list**

Moussa Sangare^1,2*^, Yaya Ibrahim Coulibaly^1,3^, Naureen Huda^4^, Silvia Vidal^5^, Sameha Tariq^6^, Michel Emmanuel Coulibaly^1^, Siaka Yamoussa Coulibaly^1^, Lamine Soumaoro^1^, Ilo Dicko^1^, Bourama Traore^1,3^, Ibrahim Moussa Sissoko^1^, Sekou Fantamady Traore^1^, Ousmane Faye^1,3^, Thomas Bruce Nutman^6^, Jesus Gilberto Valenzuela^7^, Fabiano Oliveira^7^, Seydou Doumbia^1^, Shaden Kamhawi^7^, Roshanak Tolouei Semnani^8*^

**Authors’ Affiliations**

^1^Mali International Center for Excellence in Research, Faculty of Medicine and Odonto-stomatology, University of Sciences, Techniques and Technologies of Bamako, Mali

^2^Interdisciplinary School of Health Sciences, Faculty of Health Sciences, University of Ottawa, Canada

^3^Dermatology Hospital of Bamako, Mali

^4^Department of Pediatrics, University of California, San Francisco, San Francisco, CA, USA

^5^Institut Recerca H. Sant Pau C. Sant Quintí, 77-79, Barcelona-08041, Spain

^6^Laboratory of Parasitic Diseases, LPD, National Institute of Allergy and Infectious Diseases, National Institutes of Health, Bethesda, MD, 20892, USA

^7^Vector Molecular Biology Section, LMVR, National Institutes of Health, Rockville, MD, 20852, USA

^8^Autoimmunity and Translational Immunology, Precigen, Inc. *A wholly owned subsidiary of Intrexon Corporation*, 20358 Seneca Meadows Pkwy., Germantown, MD 20876

^*^**Correspondance to**: Dr Roshanak Tolouei Semnani, email: [rsemnani@precigen.com](mailto:rsemnani@precigen.com) ;

Dr. Moussa Sangare, email: [mbsangare@icermali.org](mailto:mbsangare@icermali.org)

**Author name and address**

| **Author’s name** | **Email** |
| --- | --- |
| Moussa Sangare, MD, MPH | [mbsangare@icermali.org](mailto:mbsangare@icermali.org) |
| Yaya Ibrahim Coulibaly, MD, PhD | [yicoulibaly@icermali.org](mailto:yicoulibaly@icermali.org) |
| Naureen Huda, MD | [naureen.huda@ucsf.edu](mailto:naureen.huda@ucsf.edu) |
| Silvia Vidal, PhD | [SVidal@santpau.cat](mailto:SVidal@santpau.cat) |
| Sameha Tariq, BS | [Sameha.Tariq@gmail.com](mailto:Sameha.Tariq@gmail.com) |
| Michel Emmanuel Coulibaly, MSc | [michou@icermali.org](mailto:michou@icermali.org) |
| Siaka Yamoussa Coulibaly, MD, MPH | [yamoussa@icermali.org](mailto:yamoussa@icermali.org) |
| Lamine Soumaoro, MSc | [soumla@icermali.org](mailto:soumla@icermali.org) |
| Ilo Dicko, MD, MPH | [ilo@icermali.org](mailto:ilo@icermali.org) |
| Bourama Traore, PharmD | [bouramat@icermali.org](mailto:bouramat@icermali.org) |
| Ibrahim Moussa Sissoko, MSc | [ibrahim@icermali.org](mailto:ibrahim@icermali.org) |
| Sekou Fantamady Traore, PhD | [cheick@icermali.org](mailto:cheick@icermali.org) |
| Ousmane Faye, MD, PhD | [Faye_o@yahoo.fr](mailto:Faye_o@yahoo.fr) |
| Thomas Bruce Nutman, MD | [tnutman@niaid.nih.gov](mailto:tnutman@niaid.nih.gov) |
| Jesus G Valenzuela, PhD | [jvalenzuela@niaid.nih.gov](mailto:jvalenzuela@niaid.nih.gov) |
| Fabiano Oliveira, MD, PhD | [loliveira@niaid.nih.gov](mailto:loliveira@niaid.nih.gov) |
| Seydou Doumbia, MD, PhD | [sdoumbi@icermali.org](mailto:sdoumbi@icermali.org) |
| Shaden Kamhawi, PhD | [skamhawi@niaid.nih.gov](mailto:skamhawi@niaid.nih.gov) |
| Roshanak Tolouei Semnani, PhD | [rsemnani@precigen.com](mailto:rsemnani@precigen.com) |

**Abstract**

**Background**

In Mali, cutaneous leishmaniasis (CL) and filariasis are co-endemic. Previous studies in animal models of infection have shown that sand fly saliva enhance infectivity of *Leishmania* parasites in naïve hosts while saliva-specific adaptive immune responses may protect against cutaneous and visceral leishmaniasis. In contrast, the human immune response to *Phlebotomus duboscqi* (Pd) saliva, the principal sand fly vector in Mali, was found to be dichotomously polarized with some individuals having a Th1-dominated response and others having a Th2-biased response. We hypothesized that co-infection with filarial parasites may be an underlying factor that modulates the immune response to Pd saliva in endemic regions.

**Methodology/Principal Findings**

To understand which cell types may be responsible for polarizing human responses to sand fly saliva, we investigated the effect of salivary glands (SG) of Pd on human monocytes. To this end, elutriated monocytes were cultured in vitro, alone, or with SG, microfilariae antigen (mf ag) of *Brugia malayi*, or LPS, a positive control. The mRNA expression of genes involved in inflammatory or regulatory responses was then measured as were cytokines and chemokines associated with these responses. Monocytes of individuals who were not exposed to sand fly bites (mainly North American controls) significantly upregulated the production of IL-6 and CCL4; cytokines that enhance *leishmania* parasite establishment, in response to SG from Pd or other vector species. This selective inflammatory response was lost in individuals that were exposed to sand fly bites which was not changed by co-infection with filarial parasites.

**Conclusions/Significance**

Furthermore, infection with filarial parasites resulted in upregulation of CCL22, a type-2 associated chemokine, both at the mRNA levels and by its observed effect on the frequency of recruited monocytes. Together, our data suggest that SG or recombinant salivary proteins from Pd alter human monocyte function by upregulating selective inflammatory cytokines.

**Key words:** Filarial parasites, sand fly, salivary gland, *Phlebotomus dubosqi*, immunomodulation, Cutaneous leishmaniasis, monocytes, Mali

**Author Summary**

In Mali, cutaneous leishmaniasis (CL) and filariasis are co-endemic. We hypothesized that co-infection with helminth parasites may modulate the immune response to saliva of the CL vector *Phlebotomus duboscqi* (Pd). Hence, we investigated the effect of Pd salivary glands (SG) on human monocytes in subjects exposed or unexposed to sand fly bites in the context of a concomitant filaria infection. Monocytes of unexposed individuals selectively upregulated the production of IL-6 and CCL4 in response to SG from Pd or other vector species. In contrast, monocytes of individuals exposed to sand fly bites lost their responsiveness to SG, microfilariae antigen and LPS, irrespective of co-infection with filaria. Nevertheless, infection with filaria significantly upreguled the frequency of CCL22^+^monocytes, IL10^+^mDCs and regulatory T cells. Together, our data suggest that repeated exposure to Pd saliva alters human monocyte function towards a tolerized phenotype while co-infection with filaria favors a *Leishmania*-promoting Th2/regulatory response.

**Introduction**

Cutaneous leishmaniasis, a vector-borne disease transmitted by bites of infected phlebotomine sand flies [1], is endemic in sub Saharan Africa including Mali [2–4]. Sand flies are found in areas endemic for lymphatic filariasis (LF) that results in physical disabilities and carries an important socioeconomic impact [5]. In Mali, the major filarial parasite responsible for LF is *Wuchereria bancrofti* (Wb) but *Mansonella perstans* (Mp) is also present [6].

Sand fly saliva has an array of proteins with varied pharmacological properties that facilitate blood feeding [7]. Salivary proteins also exert immunomodulatory effects on the innate immune system [8–11] and induce a strong adaptive immunity [12–14]. Vector-transmission of *Leishmania* parasites is exclusively by the bite of infected sand flies where the parasites are co-deposited into the skin together with saliva. Studies have consistently shown that saliva generally enhances infectivity of *Leishmania* parasites in a naïve host [8] while an adaptive immune response to whole saliva or a distinct salivary protein generally protects against cutaneous and visceral leishmaniasis in rodent models of infection [15–19]. However, for certain sand fly species and certain salivary proteins induction of a specific adaptive immune response exacerbates *Leishmania* infection [19,20]. Protective salivary proteins are characterized by their induction of a Th1 delayed-type hypersensitivity response where IFN-γ, considered a strong correlate of protection in leishmaniasis, is induced. The mechanism of this protection remains to be fully elucidated but preliminary evidence points to priming of a protective anti-*Leishmania* response at the site of bite mediated by immunity to saliva [21,22]. In contrast, disease exacerbation is correlated with the induction of Th2 and/or regulatory cytokines [19]. Overall, such findings suggest that salivary proteins are potent modulators of the host immune response with consequences for the transmitted *Leishmania* parasites. While the majority of these studies were undertaken in animal models, the human immune response to saliva of *Phlebotomus duboscqi* (Pd), the principal sand fly vector in Mali, was found to be dichotomously polarized with some individuals developing a Th1-dominated response and others a Th2-biased response [23].

The altered function of the innate immune response, particularly the dysfunction of antigen presenting cells (APC), has been postulated to mediate some of the parasite-specific T cell unresponsiveness seen in patent filarial infection. Previously, we have shown that live mf of *Brugia malayi* (Bm) modulate dendritic cell (DC) function by altering the expression and function of Toll-like receptors 3 and 4, and by inducing apoptotic DC cell death [22]. In addition to the effect of live mf on DCs, we have shown that circulating monocytes from filaria-infected individuals are laden with filarial antigens, exhibit diminished antigen presentation and processing, and produce fewer proinflammatory cytokines in response to surface receptor crosslinking [24]. Therefore, we hypothesized that a filarial infection may compromise the immune response to sand fly salivary proteins, which in turn may alter the host’s immune response to *Leishmania* infection. In this study, we aimed to assess the effect of sand fly saliva on human monocytes in the context of a concomitant filarial infection.

**METHODS**

**Ethics statement**

The Institutional Review Board of the National Institutes of Health, USA, and the Ethical Committee of the Faculty of Medicine, Pharmacy and Odonto-Stomatology of Bamako, Mali approved the study. Leucopacks from health blood bank donors were obtained from the Department of Transfusion Medicine (National Institutes of Health, Bethesda, MD) under (IRB)-approved protocols (IRB# 99-CC-0168). Written informed consent was obtained from all the volunteers prior to their participation.

**Study populations**

We screened 930 individuals for *Leishmania* using the leishmanin skin test (LST); for filarial infections using the Immunochromatographic card test (ICT) for LF and thick blood smear for *Mansonella perstans* (Mp); and for antibodies to sand fly saliva by ELISA. All screened individuals were determined to be positive by sand fly antibody ELISA (SF^+^) and negative by LST test. Among those, we identified 67 SF^+^ and filaria or microfilaria-infected clinically asymptomatic (MF^+^) individuals and 61 SF^+^/MF^-^ individuals.

**Thick blood smear**

Finger-prick and venous blood samples were collected during daytime using a calibrated thick blood smear (60μl). The blood was drawn onto a microscope slide, dried and stained with 10% Giemsa using standard procedures. The stained smears were examined using a light microscope with a 10× objective for the detection of Mp microfilariae.

**ICT**

The ICT Filariasis test is a rapid-format filarial antigen test that was developed by ICT diagnostics (BinaxNow® Filariasis test, Alere Inc., Scarborough, USA). The test is designed for detection of soluble *W. bancrofti* antigens that circulate in the blood of infected humans.

**LST**

LST material is supplied as sterile 2.5ml aqueous suspensions, with each ml containing 6×10^6^ killed *L. major* promastigotes (strain MRHO/IR/75/ER) in phosphate buffered saline and 0.01% thimerosal, pH 7.0–7.1. Leishmanin (0.1ml) was injected intra-dermally in the left forearm. Readings were taken 48 to 72 hours after injection using a ball point pen to determine the induration size. Measurements with a diameter greater than 5mm were considered positive [12].

**Anti-saliva ELISA**

The blood sample made from the filter paper was obtained using a 6 mm disposable punch, eluted in 0.05% Tween PBS, and stored at room temperature (RT) overnight. The ELISA test was carried out as described previously[25]. The ELISA plates were coated with 50μl of Pd salivary gland homogenate diluted at 2μg/ml in carbonate/bicarbonate buffer (pH 9.6) overnight at 4°C. The next day the plates were washed three times with PBS- 0.05% Tween, and blocked with PBS containing 4% bovine serum albumin for 2 hours at room temperature. The plates were washed and sera (100 μl of sera (diluted 1:100 in PBS-Tween+0.5% BSA)) were added and incubated at RT for one hour. After washing, alkaline phosphatase conjugated goat anti-human IgG (Sigma, MO) was added to each well (100μl/well (1:1000 in PBS-Tween-BSA)) and incubated at 37^0^C for an hour. Following another washing, *p-nitrophenyl phosphate* substrate (Sigma Sr. Louis, MO) was added and the absorbance was read at 405nm on a Versamax microplate reader after 30 minutes. Values obtained were subtracted from those obtained for the background (where PBS replaced eluted blood).

**Mf antigen preparation**

Soluble MF Ag was made from ∼10^8^ live *Brugia malayi* MF (provided by John McCall, University of Georgia, Athens, Ga.) as described previously [26]. The MF were washed repeatedly in RPMI medium containing antibiotics and cultured overnight at 37°C in 5% CO2. Worms were harvested the following day, washed with PBS and frozen at −20°C. The frozen MF were pulverized, sonicated, and extracted in PBS at 37°C for 4 hours and then at 4°C overnight. Following centrifugation at 20000g for 30 minutes, the supernatant was passed through a 0.45μm filter and stored in aliquots at −70°C. The antigen was found to be endotoxin free by QCL-1000 kit (BioWhittaker).

**In vitro culture of monocytes**

Human monocytes were cultured at 50×10^6^ per 6-well plate in serum-free medium for 2 hours, after which the medium was removed and complete medium (RPMI 1640 medium [Lonza, Allendale, NJ] supplemented with 20 mM glutamine [Lonza], 10% heat-inactivated fetal calf serum [Gemini Bioproducts, Sacramento, CA], 100IU/ml penicillin, and 100g/ml streptomycin [Biofluids, Inc., Rockville, MD]) was added.

**In vitro cell activation**

Human monocytes were harvested and further cultured at 1×10^6^ cells/ml either in media alone, or with LPS (1μg/ml), mf antigen (10μg/ml), salivary glands from different sand fly species (1 salivary gland pair/1×10^6^ cells), salivary recombinant proteins 1H2, 4B4 and 9G11 from Pd or LJL143 from *Lutzomyia longipalpis* (2.5μg/ml), or *Leishmania* antigen (10μg/ml) for 30 minutes up to 48 hours. At the indicated time points, cells were harvested to measure mRNA expression, and the supernatants were collected to assess cytokine and chemokine production.

**RNA preparation and RT-PCR**

RNA was extracted using the RNeasy kit (Qiagen, Valencia, CA) according to the manufacturer’s instructions. cDNA generation was performed using random hexamers and reverse transcriptase (ABI, Grand Island, NY). Expression of genes (inflammatory: IP10, IL-6, CCL2, CCL4, CCL3, PDL1, and IDO; type-2 associated: CCL22; regulatory: TGF-β, IL-10) were determined by quantitative PCR using TaqMan reagents (ABI) on an ABI 7900HT. The threshold cycles (CT) for 18S and genes of interest were calculated and used to determine the relative transcript levels. The formula 1/delta CT was used to determine the relative transcript levels, where delta CT is the difference between the CT of the target gene and the CT of the corresponding endogenous 18S reference gene.

**Cytokine analysis**

The concentration of cytokines in culture supernatants was assessed using the Milliplex human cytokine/chemokine magnetic bead panel kit (EMD Millipore, Darmstadt, Germany) and the Luminex 100/200 system (Luminex Corporation, Austin, TX, USA). The lower limit of detection was 3.2pg/mL for IL-1α, IL-1β, IL-6, CCL4, IP10, CCL2, IL-10, IL5, IL-17, and IFN-γ. Supernatant cytokine concentrations were determined by ELISA for CCL22 (R&D Systems, Minneapolis, MN, USA) at a sensitivity of 12.5pg/mL according to the manufacturer’s protocol.

**Whole blood cell activation**

Whole blood was collected from SF^+^/MF^-^ and SF^+^/MF^+^ individuals and diluted with serum-free media at a 1:1 ratio and cultured in 1ml per condition: media alone, with LPS (1μg/ml), mf ag (20μg/ml), Leish ag (20μg/ml) or SG Pd (1 pair/ml) for 24 hours. Supernatants were collected and cytokine concentrations were measured using Luminex or ELISA. In parallel wells, 12 hours after stimulation, BFA (10μg/condition) was added to permeabilize cells to measure intracellular cytokines. After 24 hours, red blood cells were lysed using 1x BD lysing solution (Becton, Dickinson and Company BD Biosciences CA 95131 USA), fixed and labeled with antibodies for flow cytometry.

**Monocyte isolation from whole blood**

Peripheral blood mononuclear cells (PBMC) from the blood of Malian volunteers or blood bank donors were isolated by Ficoll diatrizoate density centrifugation [27]. Monocytes were isolated from PBMC using a CD14-negative monocyte isolation kit (Dynal Biotech, Lake Success, NY). Typically, the monocytes were ≥96% pure as assessed by flow cytometry.

**Gating strategy for flow cytometry**

Cryopreserved whole blood was thawed at room temperature, washed with RPMI 1640 plus 5mm EDTA (Life Technologies, Inc.), and diluted in RPMI 1640 supplemented with 10% FCS. Viability was determined using CD45 FITC (Becton Dickinson). Cell pellets (100μl) were incubated with anti-CD3, anti-CD4, anti-CD25, anti-CD127, anti-CD14, anti-CD19 and anti-CD16 antibodies. After 20 minutes of staining, cells were fixed using a BD FACS lysing solution (BD Biosciences). Cells were analyzed on a Fortessa flow cytometer.

**Statistical analysis**

Statistical analysis of data from real-time PCR, Luminex, and ELISA was done using a Wilcoxon matched pairs signed-rank test, and for clinical data the nonparametric Mann-Whitney test was performed to compare between SF^+^/MF^+^ and SF^+^/MF^-^ individuals using Graph Pad Prism version 8 (GraphPad Software, Inc.).

**Results**

**Saliva of the vector sand fly *Phlebotomus duboscqi* upregulates the production of CCL4 and IL-6 in human monocytes**

Recruitment and immunomodulation of monocytes at the site of a sand fly bite following exposure to saliva has been reported in the past [28]. Here, we assessed the effect of SG Pd on polarization of monocytes from healthy donors and compared it the effect of mf ag, *Leishmania* antigen (Leish ag), or LPS (positive control). To assess the phenotype of monocytes, we measured the production of cytokines associated with a proinflammatory response (IL-6, IP10, CCL2, CCL3, CCL4), a type-2 response (CCL22), and a regulatory response (IL-10). Production of the proinflammatory cytokines IL-6 and CCL4 (MIP-1-β) was significantly upregulated after stimulation for 24 hours with either SG Pd, mf ag or Leish ag (Fig 1). Interestingly, SG Pd had the most restrictive effect on monocytes, only upregulating IL-6 and CCL4. In comparison, mf ag upregulated monocytes production of all the tested cytokines (Fig 1). *Leishmania* ag also upregulated the production of all cytokines with the exception of IP10. Of note, despite the pronounced response at the protein level, mRNA expression of selected genes was not altered after 4 hours of stimulation with SG Pd, Pd recombinant salivary proteins 1H2 and 4B4, or mf ag (Supplemental Fig 1). The difference could be due to the shorter exposure to stimuli to evaluate gene expression. Further, only the mRNA levels for CCL2 and CCL4 were significantly induced by Leish ag (Supplemental Fig 1).

**Saliva of several vector species of sand flies upregulates the production of CCL4 and IL-6 in human monocytes**

As some proteins in saliva of sand fly species are species-specific, and most have been shown to possess immunomodulatory effects on T cells, macrophages and DC populations, we next examined the effect of saliva of several sand fly species on the modulation of human monocytes from healthy donors. Similar to SG Pd, saliva of *Phlebotomus papatasi* (SG Pp), *Phelobotomus argentipes* (SG Pa), *Phleobotomus. sergenti* (SG Ps), *Phelobotomus perniciosus* (SG Ppr), and *Lutzomyia longipalpis* (SG Ll) significantly upregulated the production of CCL4 and IL-6 in human monocytes (Fig 2). Additionally, CCL2 production was also significantly upregulated by SG Ll and SG Ps, while the production of a type 2 cytokine CCL22 was only enhanced by Ppr demonstrating global and species-specific effects of sand fly saliva on human monocytes (Fig 2).

**Immunomodulatory effects of salivary and parasitic antigens are lost in monocytes of individuals naturally exposed to vector bites**.

Next, we aimed to assess the response of monocytes from individuals that are naturally exposed to saliva through vector bites in the context of a helminth infection. We tested individuals in regions of Mali co-endemic for both Pd sand flies and filarial infection. The first group are individuals who are sand fly bite positive (SF^+^) and MF negative (MF^-^), hereafter referred to as SF^+^/MF^-^. The second group are individuals positive for sand fly bite (SF^+^) and positive for mf (MF^+^), hereafter referred to as SF^+^/MF^+^ (see method section for a full description). To assess cytokine production, monocytes of both groups were isolated from PBMC and cultured overnight in either media alone or with SG Pd, mf ag, or LPS (positive control).

Interestingly, stimulation with SG Pd did not induce any of the proinflammatory, type-2 or regulatory cytokines in monocytes of both groups despite having originated from individuals naturally exposed to sand fly bites (Fig 3). In fact, while IL-6 and CCL4 were upregulated in monocytes from sand fly bite-naive individuals (Fig 2), this response was lost in SF^+^ individuals regardless of their mf status (Fig 3). More importantly, exposure to sand fly bites also dampened the monocyte response to mf antigen and even to LPS, observed for some cytokines (Fig 3). Supporting previous data [24,29], monocytes of MF^+^ individuals were less responsive than monocytes of MF^-^ individuals since LPS significantly induced the production of CCL4 and IL-10 only in MF^-^ individuals (Fig 3). Of note, no significant differences between the groups were observed when basal cytokine production (media alone) was measured (Supplemental Fig 2). Collectively, these data suggest that monocytes from individuals repeatedly exposed to sand fly bites exhibit a dampened response to stimulation with any antigen.

**Increase in mRNA expression of CCL22 and frequency of CCL22^+^ monocytes in SF^+^/MF^+^ than SF^+^/MF^-^ individuals**

To assess the effect of helminth infection on gene expression, we measured the basal mRNA expression in monocytes of SF^+^/MF^-^ compared to SF^+^/MF^+^ individuals. While there was no difference in proinflammatory and regulatory cytokine production, monocytes of SF^+^/MF^+^ individuals showed a significantly higher expression of CCL22, a type-2 chemokine (Fig 4), suggesting that exposure to sand fly saliva does not change the mf-induced type-2 phenotype.

Worth to mention, whole blood collected from SF^+^/MF^+^ and SF^+^/MF^-^ individuals, cultured in media alone showed no difference in the production of any of the inflammatory, type-2 or regulatory cytokines between the groups (Supplemental Fig 3).

**SF^+^/MF^+^ individuals have a higher frequency of Treg and IL-10^+^ mDC than SF^+^/MF^-^ individuals**

To understand whether exposure to sand fly bite changes the composition of innate immune cells, we performed flow cytometry on whole blood of SF^+^/MF^-^ and SF^+^/MF^+^ individuals. To this end, we did not see a significant difference in the frequency of lymphocytes or neutrophils, but observed a significant increase in the percentage of CD25^+^/CD127^low^ Tregs in SF^+^/MF^+^ individuals (Fig 5A). Further, though there was no overall difference in the number of monocytes or mDC in SF^+^/MF^+^ as compared to SF^+^/MF^-^ individuals, a significant increase was observed in the frequency of CCL22^+^ monocytes and IL-10^+^ mDCs in SF^+^/MF^+^ as compared to SF^+^/MF^-^ individuals (Fig 5B). These data suggest that exposure to sand fly saliva through bites doesn’t change the regulatory phenotype that has already been established by helminth infection.

**Discussion/conclusion**

To date, most studies of sand fly saliva have been focused on leishmaniasis; understandable as sand flies are biological vectors that transmit *Leishmania* by bite. Nevertheless, in real-life situations humans are exposed to multiple infections. Importantly, co-infections have been shown to shape the immune response to pathogens including helminths, *Plasmodium* and *Leishmania*[30–32]. Another underexplored yet equally relevant facet to immunomodulation is that humans are frequently exposed to saliva of biting disease vectors that contains immunomodulatory and immunogenic proteins [33–35]. It has been established that sand fly saliva contains immunomodulatory molecules that manipulate the host immune response exacerbating infection by *Leishmania,* while adaptive immunity generated against salivary proteins in experimentally- or naturally-exposed hosts associates to protection from leishmaniasis [33].

Filariasis and leishmaniasis are co-endemic in Mali where the majority of inhabitants are also exposed to sand fly bites [36]. Here, we aimed at understanding how natural exposure to sand fly saliva through repeated bites, regardless of *Leishmania* infection, may affect the host response to filaria. Additionally, we also explored how filaria-infected individuals modulate the response to sand fly bites.

As monocytes are recruited to the bite site of disease vectors [31,34], we focused the investigation on understanding the response of these cells in humans that are unexposed or naturally-exposed to sand flies. Interestingly, monocytes recovered from individuals naturally-exposed to sand fly bites were refractory to antigenic challenge, and became unresponsive to filarial or leishmanial antigens, or even potent activators such as LPS. This finding has important implications regarding the response of bite-experienced humans to various pathogens. This is also of particular relevance to *Leishmania* parasites that are transmitted by sand flies since monocytes are their host cells. The effect of monocyte dysfunction upon repeated exposure to sand fly bites on the establishment and progression of *Leishmania* infection remains to be assessed.

In contrast to saliva-exposed monocytes, cells from healthy volunteers produced significantly higher levels of several inflammatory and regulatory cytokines/chemokines in response to filarial and leishmanial antigens but only upregulated the production of CCL4 (MIP-1 β) and IL-6 in response to sand fly saliva. Our findings support previous studies that reported the induction of IL-6 and CCL2, another monocyte chemoattractant, in response to sand fly saliva or bites [33][31]. IL-6 is a proinflammatory cytokine that has been shown to down modulate the killing of *Leishmania amazonensis* in human macrophages [37]. In addition, it has been shown that *Lutzomyia longipalpis* sand fly salivary gland homogenates enhance IL-6 production by human monocytes which may enhance the survival of *Leishmania* in humans infected with the parasite [38]. A study reporting the upregulation of CCL4 by *Leishmania donovani* hypothesized that this parasite induces the expression of CCL4 in host cells to attract regulatory CCR5+ cells to the site of infection, and to further downregulate host immune response [39]. Interestingly, similar to SG Pd, Leish ag, and LPS also upregulate CCL4 in unexposed monocytes, but not if monocytes have been pre-exposed to sand fly bites. This data suggests that repeated exposure to sand fly bites in endemic regions alters the innate immune response rendering them less responsive to further activation with either sand fly salivary proteins, *Leishmania* antigens or LPS.

Chronic infection with filarial parasites is known to bias the human immune response towards a more regulatory Th2/M2 profile, hence diminishing the Th1/M1 response[32][40]. In this study we have observed that compared to cells from MF^-^/SF^+^ subjects, monocytes from MF^+^/SF^+^ individuals produce less cytokines and are more of the M2-phenotype, evidenced by a higher CCL22 mRNA expression and a higher frequency of CCL22^+^ cells. Furthermore, the frequency of IL-10^+^ myeloid DC and T regulatory cells are increased in MF^+^/SF^+^ as compared to MF^-^/SF^+^ individuals, indicative of an immune response biased towards a regulatory state. Further mf ag induced the production of type-1, type 2, and regulatory cytokines in monocytes from individuals not previously exposed to sand fly bites. However, in individuals that are exposed to sand fly bites in endemic regions, microfilariae (SF^+^/MF^+^) does not alter basal cytokine production from either monocytes or whole blood. This is in accordance with the helminth induction of both regulatory and type-2 phenotypes [41] which seems not to be affected by exposure to sand fly bites.

This study demonstrates that repeated exposure to sand fly saliva has a profound effect on human monocytes that renders them unresponsive to stimuli. Superimposed on this, the immune response of individuals residing in regions co-endemic for filaria is further modulated by microfilariae, revealing the complexity of the immune interactions that shape the human response to disease vectors and the pathogens they transmit in real-life scenarios. Studies that consider multi-factorial settings that affect the human immune system simultaneously are much needed and may reveal new information vital to understanding how diseases establish in the natural environment.

**Acknowledgments**

The authors would like to thank the study communities in Kolokani and Kolondieba and the Fieldworkers for their support. We also would like to thank the ICER community and field workers for their dedication and support.

**References:**

1. CDC - Centers for Disease Control and Prevention; CDC - Leishmaniasis [Internet]. Parasites - Leishmaniasis. 2018 [cited 2020 Aug 27]. Available from: https://www.cdc.gov/parasites/leishmaniasis/

2. Alvar J, Vélez ID, Bern C, Herrero M, Desjeux P, Cano J, et al. Leishmaniasis worldwide and global estimates of its incidence. Kirk M, editor. PLoS One. 2012. p. e35671.

3. Boakye DA, Wilson M, Kweku M. A review of leishmaniasis in west Africa. Ghana Med J. Ghana Medical Association; 2005;39:94–7.

4. Oliveira F, Doumbia S, Anderson JM, Faye O, Diarra SS, Traoré P, et al. Discrepant prevalence and incidence of Leishmania infection between two neighboring villages in Central Mali based on leishmanin skin test surveys. Louzir H, editor. PLoS Negl Trop Dis. 2009;3:e565.

5. Eppig C, Fincher CL, Thornhill R. Parasite prevalence and the worldwide distribution of cognitive ability. Proc R Soc B Biol Sci. 2010. p. 3801–8.

6. Dembélé M, Bamani S, Dembélé R, Traoré MO, Goita S, Traoré MN, et al. Implementing preventive chemotherapy through an integrated national neglected tropical disease control program in Mali. PLoS Negl Trop Dis. 2012;6.

7. Ribeiro JMC, Francischetti IMB. R OLE OF A RTHROPOD S ALIVA IN B LOOD F EEDING : Sialome and Post-Sialome Perspectives. Annu Rev Entomol. 2002;48:73–88.

8. Titus RG, Bishop J V, Mejia JS. The immunomodulatory factors of arthropod saliva and the potential for these factors to serve as vaccine targets to prevent pathogen transmission. Parasite Immunol. 2006. p. 131–41.

9. Brodie TM, Smith MC, Morris R V, Titus RG. Immunomodulatory effects of the Lutzomyia longipalpis salivary gland protein maxadilan on mouse macrophages. Infect Immun. 2007;75:2359–65.

10. Lima EP, Goulart MOF, Rolim Neto ML, Sommerfeld J, Kroeger A, Frentiu F, et al. Meta-analysis of studies on chemical, physical and biological agents in the control of Aedes aegypti. BMC Public Health. BioMed Central; 2015;15:858.

11. Wheat WH, Pauken KE, Morris R V, Titus RG. Lutzomyia longipalpis salivary peptide maxadilan alters murine dendritic cell expression of CD80/86, CCR7, and cytokine secretion and reprograms dendritic cell-mediated cytokine release from cultures containing allogeneic T cells. J Immunol. 2008;180:8286–98.

12. Kamhawi S, Belkaid Y, Modi G, Rowton E, Sacks D. Protection against cutaneous leishmaniasis resulting from bites of uninfected sand flies. Science. 2000;290:1351–4.

13. Oliveira F, Jochim RC, Valenzuela JG, Kamhawi S. Sand flies, Leishmania, and transcriptome-borne solutions. Parasitol Int. 2009;58:1–5.

14. Bethony JM, Cole RN, Guo X, Kamhawi S, Lightowlers MW, Loukas A, et al. Vaccines to combat the neglected tropical diseases. Immunol Rev. 2011;239:237–70.

15. Oliveira F, Lawyer PG, Kamhawi S, Valenzuela JG. Immunity to distinct sand fly salivary proteins primes the anti-leishmania immune response towards protection or exacerbation of disease. Lehane MJ, editor. PLoS Negl Trop Dis. 2008;2:e226.

16. Anderson JM, Samake S, Jaramillo-Gutierrez G, Sissoko I, Coulibaly CA, Traoré B, et al. Seasonality and prevalence of Leishmania major infection in Phlebotomus duboscqi neveu-lemaire from two neighboring villages in Central Mali. Bates PA, editor. PLoS Negl Trop Dis. 2011;5:e1139.

17. Aday LA. Health status of vulnerable populations. Annu Rev Public Health. 1994;15:487–509.

18. Morris R V, Shoemaker CB, David JR, Lanzaro GC, Titus RG. Sandfly Maxadilan Exacerbates Infection with Leishmania major and Vaccinating Against It Protects Against L. major Infection. J Immunol. 2001;167:5226–30.

19. de Moura TR, Oliveira F, Rodrigues GC, Carneiro MW, Fukutani KF, Novais FO, et al. Immunity to Lutzomyia intermedia saliva modulates the inflammatory environment induced by Leishmania braziliensis. PLoS Negl Trop Dis. 2010;4:e712.

20. de Moura TR, Oliveira F, Novais FO, Miranda JC, Clarêncio J, Follador I, et al. Enhanced Leishmania braziliensis infection following pre-exposure to sandfly saliva. Jaffe C, editor. PLoS Negl Trop Dis. 2007;1:e84.

21. Gomes R, Teixeira C, Teixeira MJ, Oliveira F, Menezes MJ, Silva C, et al. Immunity to a salivary protein of a sand fly vector protects against the fatal outcome of visceral leishmaniasis in a hamster model. Proc Natl Acad Sci. 2008;105:7845–50.

22. Rossi M, Fasel N. How to master the host immune system? Leishmania parasites have the solutions! Int Immunol. Oxford University Press; 2018;30:103–11.

23. Oliveira F, de Carvalho AM, de Oliveira CI. Sand-fly saliva-Leishmania-man: The trigger trio. Front Immunol. Frontiers Media SA; 2013;4:375.

24. Semnani RT, Keiser PB, Coulibaly YI, Keita F, Diallo AA, Traore D, et al. Filaria-induced monocyte dysfunction and its reversal following treatment. Infect Immun. American Society for Microbiology (ASM); 2006;74:4409–17.

25. Traoré B, Oliveira F, Faye O, Dicko A, Coulibaly CA, Sissoko IM, et al. Prevalence of Cutaneous Leishmaniasis in Districts of High and Low Endemicity in Mali. Boelaert M, editor. PLoS Negl Trop Dis. 2016;10:e0005141.

26. Steel C, Nutman TB. Helminth antigens selectively differentiate unsensitized CD45RA+ CD4+ human T cells in vitro. J Immunol. 1998;160:351–60.

27. Byum A. Isolation of mononuclear cells and granulocytes from human blood. Isolation of monuclear cells by one centrifugation, and of granulocytes by combining centrifugation and sedimentation at 1 g. Scand J Clin Lab Investig Suppl. 1968;97:77–89.

28. Menezes MJ, Costa DJ, Clarêncio J, Miranda JC, Barral A, Barral-Netto M, et al. Immunomodulation of human monocytes following exposure to Lutzomyia intermedia saliva. BMC Immunol. 2008;9:12.

29. Sasisekhar B, Aparna M, Augustin DJ, Kaliraj P, Kar SK, Nutman TB, et al. Diminished monocyte function in microfilaremic patients with lymphatic filariasis and its relationship to altered lymphoproliferative responses. Infect Immun. American Society for Microbiology (ASM); 2005;73:3385–93.

30. Shen SS, Qu XY, Zhang WZ, Li J, Lv ZY. Infection against infection: Parasite antagonism against parasites, viruses and bacteria. Infect Dis Poverty. BioMed Central Ltd.; 2019;8.

31. Dey R, Joshi AB, Oliveira F, Pereira L, Guimarães-Costa AB, Serafim TD, et al. Gut Microbes Egested during Bites of Infected Sand Flies Augment Severity of Leishmaniasis via Inflammasome-Derived IL-1β. Cell Host Microbe. Cell Press; 2018;23:134-143.e6.

32. Damania B, Dittmer DP. What lies within: Coinfections and immunity. Cell Host Microbe. Cell Press; 2014. p. 145–7.

33. Lestinova T, Rohousova I, Sima M, de Oliveira CI, Volf P. Insights into the sand fly saliva: Blood-feeding and immune interactions between sand flies, hosts, and Leishmania. PLoS Negl Trop Dis. Public Library of Science; 2017;11.

34. Pingen M, Schmid MA, Harris E, McKimmie CS. Mosquito Biting Modulates Skin Response to Virus Infection. Trends Parasitol. Elsevier Ltd; 2017. p. 645–57.

35. Manning S, Danielson KM. The immunomodulatory role of tumor-derived extracellular vesicles in colorectal cancer. Immunol. Cell Biol. John Wiley and Sons Inc.; 2018. p. 733–41.

36. Sangare MB, Coulibaly YI, Coulibaly SY, Coulibaly ME, Traore B, Dicko I, et al. A cross-sectional study of the filarial and Leishmania co-endemicity in two ecologically distinct settings in Mali. Parasites and Vectors. BioMed Central Ltd.; 2018;11:18.

37. Hatzigeorgiou DE, He S, Sobel J, Grabstein KH, Hafner A, Ho JL. IL-6 down-modulates the cytokine-enhanced antileishmanial activity in human macrophages. J Immunol. 1993;151:3682–92.

38. Tomiotto-Pellissier F, Bortoleti BT da S, Assolini JP, Gonçalves MD, Carloto ACM, Miranda-Sapla MM, et al. Macrophage Polarization in Leishmaniasis: Broadening Horizons. Front Immunol. NLM (Medline); 2018;9:2529.

39. Kumari S, Shivam P, Kumar S, Jamal F, Singh MK, Bimal S, et al. Leishmania donovani mediated higher expression of CCL4 induces differential accumulation of CD4+CD56+NKT and CD8+CD56+NKT cells at infection site. Cytokine. Academic Press; 2018;110:306–15.

40. Metenou S, Nutman TB. Regulatory T cell subsets in filarial infection and their function. Front Immunol. Frontiers Media SA; 2013;4.

41. Semnani RT, Mahapatra L, Moore V, Sanprasert V, Nutman TB. Functional and phenotypic characteristics of alternative activation induced in human monocytes by interleukin-4 or the Parasitic nematode Brugia malayi. Infect Immun. American Society for Microbiology (ASM); 2011;79:3957–65.

**Additional Information**

**Financial Disclosure Statement**

This work was supported by the National Institutes of Health under Award Number P50AI098505 (Tropical Medicine Research Center, TMRC) through the Division of extramural Research Program of NIAID, National Institutes of Health, Bethesda, MD, USA (<https://www.nih.gov/>). “The funders had no role in study design, data collection and analysis, decision to publish, or preparation of the manuscript;

**Competing interests**

The authors have declared that no competing interests exist.

**Author contributions**

MS, YIC, RTS, TBN, SK, FO, JGV, OF, SD and SFT conceived and designed the study. MS YIC, NH, SV, FO, RTS, TBN, OF, SYC, BT, SD, MEC, SS, ST and MIS collected the data and performed the experiments in the laboratory. MS, YIC, ID, RTS, FO, SD, and SFT analyzed the data. TBN, RTS, FO, JGV, SD, SK and SFT contributed reagents, materials, analysis tools. MS, RTS, SK, YIC, SYC, TBN, SD and FO managed the data and drafted the paper, did the statistical analysis and helped to draft the manuscript. All authors read and approved the final manuscript.
