# Supplementary material for: Individuals co-exposed to sand fly saliva and filarial parasites exhibit altered monocyte function
Source: PLoS Negl Trop Dis. 2021 Jun 9;15(6):e0009448. doi: 10.1371/journal.pntd.0009448 (PMC8189443; doi:10.1371/journal.pntd.0009448)
Supplement: Attachment — Submitted filename: Reviewer response Moussas paper final.docx [file pntd.0009448.s006.docx]

**Reviewer #1: Overall, there seem to be very large differences in basal cytokine/chemokine expression of monocytes from the non-exposed (mainly North American, Fig. 1) versus the endemic individuals (SG+/MF+ and SG+/MF-, Fig. 2). Looking specifically at basal IL-6 and CCL4 levels, these fluctuate between 1 and 200 pg/mL in non-exposed individuals versus 100-20,000 pg/mL in individuals from Mali. This is a 100-fold difference in basal cytokine levels, which is neither discussed nor attempted to be explained in the manuscript.**

We agree with the reviewer that there is indeed a large difference in the basal levels of cytokines between north Americans (Fig 1) and endemic individuals from Mali (Fig 3). We have now added a sentence in the result section on page 13 to address this:

*“Collectively, these data suggest that monocytes from individuals repeatedly exposed to sand fly bites exhibit a dampened response to further stimulation. This could be due to a much higher basal cytokine expression in monocytes of individuals that are naturally exposed to sand fly bite (Fig 3; closed circles) than unexposed individuals from North America (Fig 1; closed circles).”*

**The statement in lines 283-284 is misleading as it only refers to SG+/MF+ and SG+/MF-.**

As well, we have now clarified the statement: line 342 in the revised manuscript:

*“Of note, no significant difference between the MF^+^/SF^-^ and MF^-^/SF^+^ individuals were observed when basal cytokine production (media alone) was measured (Supplemental Fig 2).*

**On the background of the very high baseline cytokine levels, I do not agree with the conclusion or concept that the cells would have become unresponsive or dysfunctional due to sand fly exposure. The cells seem to be already strongly activated for one or another reason with only a high LPS concentration still having some limited additional stimulatory effect.**

We agree with the reviewer and has modified our wording accordingly. Please see page 13 for this modification.

*“Collectively, these data suggest that monocytes from individuals repeatedly exposed to sand fly bites exhibit a dampened response to further stimulation. This could be due to a much higher basal cytokine expression in monocytes of individuals that are naturally exposed to sand fly bite (Fig 3; closed circles) than unexposed individuals from North America (Fig 1; closed circles).”*

**Moreover, how can authors attribute differences in monocyte responses to prior sand fly exposure per se in the absence of an SG- endemic control group (which could not be identified in the study area as all individuals are SG+).**

Since majority of individuals living in the endemic areas in Mali have been exposed to Sand fly bite we don’t have any access to SF negative control from the same regions of Mali. Therefore, our comparison is to North American individuals that have not been exposed to sand fly bite. We agree that there may be other factors involved for this highly activated cells, and we have to consider those differences between the endemic regions and North America. To draw attention to this, we have added the following paragraph to our discussion:

*“It is important to note that monocytes from Malian individuals produce a higher basal level of cytokines compared to controls, rendering them less responsive to antigen-specific stimulation. However, since the majority of individuals living endemic areas in Mali have been exposed to sand fly bites, we compared the response of their monocytes to cells from North American individuals as controls. As such, factors other than repeated exposure to sand fly saliva may have contributed to the induction of the heightened state of activation observed for Malian monocytes.”*

**As the authors have determined the anti-Pd SG antibody titers as indicators of sand fly exposure, could any correlations be found with monocyte responsiveness? This is probably one of the only options to make a link with sand fly exposure.**

Thank you for your suggestion and now we have done correlations (ab titer versus basal cytokine expression of all SF^+^ individuals regardless of MF status) please see below. Our data suggests that there is no significant correlation between any of the tested cytokines and antibody titers. Nevertheless, the cellular immune response may be very different from the antibody response. Due to the absence of a clear conclusion, we will keep our discussion paragraph pointing out the possibility that other factors may have contributed to the induction of this heightened state of activation as mentioned above.

**Where were the Malian individuals recruited?**

We screened 930 individuals in southern districts of Mali particularly in Kolondieba (village of Boundioba) and Kolokani (villages of Tieneguebougou and Bougoudiana). We have shown in the past that these areas of Mali are co-endemic for filarial and Leishmania infections (ref # 25). This is now added to our method section.

**How was the blood anticoagulated and cryo-preserved?**

In the field, blood was collected in EDTA tubes, packaged in cryoboxes and transported to Bamako in air-conditioned 4x4 vehicles (~ 4 hour drive). In the laboratory, samples were processed as soon as they were received (serum collection, separation of PBMCs, monocytes isolation, culture and activation).

**For improved reading flow, monocyte isolation from whole blood (214-2018) may be moved above the section on in vitro culture.**

Thank you for the suggestion. We have now moved the monocyte isolation and whole blood above the section on in vitro culture. As well, we clarified the isolation of monocytes from healthy volunteers and isolation of monocytes from whole blood of MF^+^/SF^+^ and MF^-/^SF^+^ individuals. Please see our revised method section.

**After monocyte isolation, how long were monocytes cultured prior to antigenic stimulation. Was this always the same?**

Monocytes were cultured at a final concentration of 1^e6^/1ml of media. While the cell number might have been different but the final concentration for activation always remained the same. Post isolation, monocytes were activated with antigen and other stimuli as described in our method section.

***“Monocyte isolation from whole blood of MF^+^/SF^+^ and MF^-/^SF^+^ individuals***

*Peripheral blood mononuclear cells (PBMC) from the blood of Malian volunteers or blood bank donors were isolated by Ficoll diatrizoate density centrifugation [27]. Monocytes were isolated from PBMC using a CD14-negative monocyte isolation kit (Dynal Biotech, Lake Success, NY). Typically, the monocytes were ≥96% pure as assessed by flow cytometry.*

***Monocyte activation from MF^+^/SF^+^ and MF^+^/SF^-^ individuals***

*Monocytes isolated using CD14-negative isolation kit (Dynal Biotech) were cultured at 1×10^6^ cells/ml either in media alone, or with LPS (1μg/ml), MF antigen (10μg/ml), salivary glands from different sand fly species (1 salivary gland pair/1×10^6^ cells), Leishmania antigen (10μg/ml) for 24 hours. At the indicated time points, cells were harvested and the supernatants were collected to assess cytokine and chemokine production.”*

**The authors indicate in the method section that antigen exposure was for 30min up to 48h (lines 185-186), but only 24h data are reported and a single experiment with a short 40min exposure for a transcriptional study.**

We have exposed monocytes to ag stimulation for 30 min, 4 hrs, 24 hrs, and 48 hrs. The interesting time point for cytokine change was at 24 hours (Figure 1; n=6-13; 6-13 independent monocyte donors). As well, we have looked at the mRNA levels at an earlier time point (4 hrs for gene expression) as reported in the supplemental Figure 1, with n=5-7 (in 5-7 independent monocyte donors). We have now clarified the activation time points in our method section.

**The choice of the recombinant salivary proteins, information on their putative functions and references to the methods of production/purification are lacking in the manuscript. Was the Leishmania antigen always from L. major, this is not clear from the methods section and the various figures.**

The sand fly salivary proteins were chosen based on their observed immunogenicity in humans (Marzouki et al., 2012; Cecilio et al., 2017; Guimaraes-Costa et al., 2021). The recombinant protein 1H2 (Pdum34; accession no. DQ834330) is a PpSP32-like silk-related protein, while 4B4 (Pdum35; accession no. DQ826522) and 4C2 (Pdum10; accession no. DQ826519) belong the yellow family of proteins from *Phlebotomus duboscqi* saliva (Kato et al, 2006). The function of silk-related proteins remains unknown but yellow proteins have biogenic amine binding properties (Sima et al., 2016; Sumova et al., 2019). Additionally, 4B4 and 4C2 have been recently shown to act as neutrophil chemoattractants (Guimaraes-Costa et al., 2021). LJL143 (accession no. AY445936) from saliva of *Lutzomyia longipalpis* has both anticoagulant (Collin et al., 2012) and anti-complement (Mendes-Sousa et al., 2017) activities. Briefly, cDNA encoding for each of the target salivary proteins from *Ph. duboscqi* and *Lu. longipalpis* was cloned into the VR2001-TOPO vector, expressed in HEK-293F cells, and purified using HPLC as previously described (Abdeladhim et al., 2016).

*Leishmania* antigen, kindly donated by Dr. David L. Sacks (NIAID/NIH) was prepared by freeze/thaw cycles of *L. major* stationary-phase promastigotes as described previously (Charmoy et al., 2016). This is now added to the references.

This is now added to the method section.

**Can the authors discuss the choice and potential limitations of Brugia malayi antigen as filarial antigen in the stimulation assays for a study area where other filarial species occur (Wuchereria, Mansonella), i.e. how comparable are the antigenic repertoires.**

It will be extremely difficult to ascertain the antigenic repertoires for *W. bancrofti* and *M. perstans* because of the non-availability of parasite material. Since *B. malayi* is the only one that can be maintained in an animal model and which resembles *W. bancrofti* at the predicted proteome; it is likely to have a similar antigenic repertoire. As well, all major diagnostic and vaccine candidates for *W.* bancrofti have orthologues in *B. malayi*.

**In figure 1, it is not clear why so few data points were reported for IL-10 as compared to the other cytokines for the SG Pd stimulated condition. Are data points lacking or are they below detection limit? The legend indicates that each line represents an independent donor (n= 6-8). Based on the number of lines, a quick check seems to indicate that there are up to 14 donors in most groups.**

Thank you for pointing that out.

1) All cytokines: mf activation (n=6)
2) All cytokines: LPS (n=14); Leish ag (n=14); SG PD (n=14, except for IL-10; n=8)

We have changed that in our legend to n=6-14

**I understand that flow cytometry was done on stimulated/fixed cells of whole blood and on cryopreserved blood. I assume that the same antibody panels were used. Information about the reagents used for intracellular staining seems to be lacking. It is also not entirely clear how the mDCs were identified based on the described panel in lines 223-224. It is clear that additional markers were needed (CD11c+, CD123-, HLA-DR+). For clarity, can authors also indicate the markers used to identify the Tregs and differentiate them from activated T cells. Based on the used panel, I assume that authors can include the B cell frequency as additional information in figure 5 as these also may exert regulatory functions.**

We appreciate the comments. Some information about the antibodies for the staining was missing and we have now included all the antibodies and the gating strategy for mDCs and Tregs in the method section.

***“Staining and Gating strategy for flow cytometry***

*Cryopreserved whole blood was thawed at room temperature, washed with RPMI 1640 plus 5mm EDTA (Life Technologies, Inc.), and diluted in RPMI 1640 supplemented with 10% FCS Viability was determined using CD45 FITC (Becton Dickinson). Cell pellets (100μl) were incubated with different panels of antibodies including anti-CD3-Alexa Fluor700, anti-CD4-APC, anti-CD127-PE-Cy5 (Bio Legend), anti-CD25-APC-Cy7, anti-CD14-APC-Cy7, anti-CD15-PE-Cy5, anti-CD19-PE-Cy7, anti-CD11c PE-Cy5, anti-HLA-DR-APC-Cy7, anti-CD123-PE-Cy7 (BD Biosciences) and anti-CD16-Pacific Orange (Invitrogen). In those panels to determine the production of chemokines, we also include anti-CCL22-PE and anti-IL-10- Pacific Blue (BD Biosciences). After 20 minutes of staining, cells were fixed using a BD FACS lysing solution (BD Biosciences). Cells were analyzed on a Fortessa flow cytometer.*

*Tregs were analyzed gating on CD3+ CD4^+^ CD25^+^ CD127^low^ and mDCs were analyzed gating on Lineage (CD3, CD14, CD20-FITC) negative HLA-DR^+^CD11c^+^CD123^+ “^*

B cell frequency was not different in figure 5. However, results have been included in the legend.

***Fig 5. SF^+^/MF^+^ individuals have a higher frequency of Treg than SF^+^/MF^-^ individuals.***

*Whole blood from SF^+^/MF^+^ (n=5) and SF^+^/MF^-^ (n=5) individuals were RBC-lysed and the frequency of different subsets were measured by flow cytometry.* *Frequency of B lymphocytes were not different between SF^+^/MF^-^ and SF^+^/MF^+^ (1.81±1.21 and 3.96±2.38 respectively, p=0.11). Statistical difference in frequency of subsets between the two groups are measured using nonparametric Mann- Whitney; * (p<0.05).*

**Abstract: the first sentence of the conclusions/significance section seems to fit in the above paragraph.**

We agree with the reviewer and changed the first sentence to the above paragraph in our abstract. **Line 165: what is meant by “eluted” blood**

We have clarified this in the method section.

*“The blood sample made from the filter paper was obtained using a 6 mm disposable punch, eluted in 0.05% Tween PBS, and stored at room temperature (RT) overnight. The serum obtained from this process is referred to as “eluted blood”. The ELISA test was carried out as described previously [26].”*

**IP-10 and the abbreviation for microfilariae is not consistent (capitalization) throughout text and figures.**

Thank you for pointing that out. We have changed all abbreviations to be consistent throughout the text and figures. **Greek letters were not readable in figures 4 and supplemental figure 3.**

We have modified the quality of our figures.

Reviewer #2: (No Response)
